# Delayed reconstitution of B cell immunity to pneumococcus in HIV-infected Malawian children on antiretroviral therapy

**DOI:** 10.1016/j.jinf.2014.10.011

**Published:** 2015-06

**Authors:** Oluwadamilola H. Iwajomo, Peter Moons, Rose Nkhata, David Mzinza, Abiodun D. Ogunniyi, Neil A. Williams, Robert S. Heyderman, Adam Finn

**Affiliations:** a School of Cellular and Molecular Medicine, University of Bristol, Bristol, United Kingdom; b Malawi Liverpool Wellcome Trust Clinical Research Programme, University of Malawi College of Medicine, Blantyre, Malawi; c Department of Pediatrics, University of Malawi College of Medicine, Blantyre, Malawi; d Research Centre for Infectious Diseases, School of Molecular and Biomedical Science, The University of Adelaide, Adelaide, Australia

**Keywords:** HIV, Antiretroviral therapy, Children, Malawi, Pneumococcal infection, Memory B cells, ELISPOT

## Abstract

**Objective:**

Despite CD4^+^ count restoration and viral load suppression with antiretroviral therapy (ART), HIV-infected children remain at increased risk of life-threatening infections including invasive pneumococcal disease (IPD). We therefore investigated whether persistent susceptibility to IPD following ART is associated with incomplete recovery of B-cell function.

**Methods:**

41 HIV-infected Malawian children commencing ART were followed-up for a 1 year period during which time blood samples were collected at 0, 3, 6 and 12 months for comprehensive immunophenotyping and pneumomococcal-specific Memory B-cell Enzyme-Linked Immunospot assays. In addition, nasopharyngeal swab samples were cultured to determine pneumococcal carriage rates.

**Results:**

Normalization of major lymphocyte subsets such as CD4^+^ percentages was evident following 3 months of ART. The proportions of mature naïve B cells (CD19^+^ CD10^−^ CD27^−^ CD21^hi^) and resting memory B cells (CD19^+^ CD27^+^ CD21^hi^) increased and apoptosis-prone mature activated B cells (CD19^+^ CD21^lo^ CD10^−^) decreased markedly by 12 months. However, in the context of high nasopharyngeal pneumococcal carriage rates (83%), restoration of pneumococcal protein antigen-specific B-cell memory was more delayed.

**Conclusions:**

These data show that, in chronically HIV-infected children receiving ART, improvement in B-cell memory profiles and function is slower than CD4^+^ T-cells. This supports early initiation of ART and informs research into optimal timing of immunization with pneumococcal vaccines.

## Highlights

• HIV-infected children on ART have delayed recovery of pneumococcal B-cell memory.
• Pneumococcal carriage remained high during ART.
• Mature naïve and resting memory B cells increased after 3 months of ART.
• Apoptosis prone B cell proportions reduced during ART to levels seen in controls.

## Introduction

*Streptococcus pneumoniae* is a leading cause of infectious death and hospitalization in HIV-infected adults and children in most African countries.^1,2^ Antiretroviral therapy (ART) leads to a reduction in the incidence of invasive pneumococcal disease (IPD) but the risk remains high.^3–6^ It is widely proposed that defective T-cell mediated immunity may be responsible for this disease burden,^7–9^ however, we have recently shown that compared to healthy uninfected children, even minimally symptomatic HIV-infected individuals with preserved CD4^+^ percentage have an overrepresentation of mature activated B cells, suggestive of immune activation and apoptosis, and low numbers of pneumococcal protein antigen–specific memory B cells.^10^ For at least two decades, the peripheral blood CD4^+^ T cell count or percentage in young children has been used as a correlate of HIV disease progression both as an indicator for the commencement of ART and to monitor its effectiveness when used.^11,12^ Effective treatment with ART leads to normalization of the CD4^+^ T cell count in blood associated with reduction in levels of inflammation, redistribution of T cells between tissues and blood, decrease in cell turn over and increase in thymic productivity and therefore CD4^+^ T-cell function.^13–17^ Moir and colleagues have reported that despite adequate CD4^+^ count recovery with ART, chronically infected adults have poor B cell memory functional profiles in response to HIV and non-HIV antigens when compared to individuals receiving ART with more recent infection.^18^ We therefore hypothesized that the persistent susceptibility to IPD seen in African children receiving ART may be explained by poor recovery of B-cell function and consequent delay in the re-establishment of natural immunity to *S. pneumoniae*.

Accordingly we prospectively investigated children with vertically acquired HIV infection commencing ART in Malawi where there is a high burden of IPD.^19^ We demonstrate that normalization of the circulating B cell phenotype occurs rapidly following the initiation of ART but that, in the context of high nasopharyngeal pneumococcal carriage rates, reconstitution of pneumococcal protein antigen-specific B cell memory is slower.

## Patients, materials and methods

### Study participants

Following written informed consent from parents or guardians, 45 HIV-infected children eligible to commence ART according to the Malawi National ART program guidelines operating at the time of enrollment, based either on clinical criteria (WHO pediatric stage 3 or 4) or on low CD4^+^ count or percentage^20^ were recruited at Queen Elizabeth Central Hospital (Blantyre, Malawi), a large district and tertiary referral hospital. A maximum of 5 ml whole blood and a nasopharyngeal swab sample were collected from each study participant on enrollment. In order to exclude malaria as a confounding factor in the immunological assays, all children were tested for malaria by microscopic examination of blood films. Children were monitored for a 1 year period following commencement of ART, during which time blood samples were collected at 0, 3, 6 and 12 months, while nasopharyngeal swab samples were collected monthly for the first 6 months, and then every 2 months thereafter. None of the participants received any pneumococcal vaccine before or during the study. This study complies with relevant guidelines and institutional practices of the Malawi-Liverpool Wellcome Trust Clinical Research Programme and the University of Malawi College of Medicine and was approved by the College of Medicine Research Ethics Committee (P.11/07/591).

Thirty-seven HIV-uninfected controls within the same age range undergoing elective surgery at the same hospital were recruited as part of a separate contemporaneous study reported elsewhere.^10^ The median values for pneumococcal carriage and major phenotypic parameters, from these children were used for comparative purposes.

### Carriage study and bacterial culture

Nasopharyngeal samples were collected from study participants using a swab (Medical wire) held by the shaft with the tip inserted into the anterior nares and the medial nasal wall carefully followed till resistance was felt. The swab was then rotated through 180° on its long axis to ensure good mucosal contact and withdrawn. Swabs were inoculated into 1.5 ml skim milk-tryptone-glucose-glycerin broth (STGG) and frozen.^21^ After storage and thawing, 50 μl of broth was subsequently inoculated onto sheep blood agar containing 5 μg/ml gentamicin. *S. pneumoniae* was identified by alpha hemolysis, colony morphology, bile salt solubility and optochin sensitivity.^22^

### Immunophenotyping

The proportions and absolute numbers of B and T cells were estimated in EDTA whole blood samples by flow cytometry using the following antibodies: fluorescein isothiocyanate (FITC)-labeled anti-CD19 & anti-CD21; phycoerythrin (PE)-labeled anti-CD8, anti-CD27 & anti-IgD; peridinin chlorophyll protein (PerCP)-labeled CD3 & anti-CD19; allophycocyanin (APC)-labeled anti-CD4, anti-CD10 & anti-CD27. All antibodies used in flow cytometry assays were obtained from BD Biosciences Ltd, with the exception of anti-CD21 (Beckman Coulter). B-cell subtypes were characterized using surface markers described by Moir and colleagues.^18,23^ Whole blood was incubated with respective antibodies for 20 min at room temperature in the dark. The red blood cells were lysed for 30 min using 1x lysis solution (BD). The white blood cells were then pelleted by centrifugation (450 *g*, 30 min, 25 °C), washed in phosphate buffered saline (PBS) supplemented with 0.5% bovine serum albumin (Sigma) and fixed with 2% paraformaldehyde (Sigma) before acquisition on a flow cytometer. At least 100,000 events were acquired within the lymphocyte gate using CellQuest Pro software on a four-color flow cytometer (BD FACSCalibur, BD Biosciences) or the Summit software version 4.3 on a CyAn ADP (Beckman Coulter). Lymphocytes were gated using forward and side scatter characteristics. Results were analyzed using FlowJo software version 7.2.2 (Tree Star Inc., San Carlos, CA).

### Memory B cell Enzyme-Linked Immunospot (ELISPOT) assay

Polyclonal stimulation was used to induce differentiation of memory B cells into antibody secreting cells (ASC) *in vitro*.^24^ Pneumococcal specific ASC were then enumerated using an ELISPOT assay. Briefly, peripheral blood mononuclear cells (PBMC) were isolated by density gradient centrifugation using Lymphoprep (Axis Shield plc), resuspended in complete RPMI medium (RPMI-1640 supplemented with 10 mM HEPES, 100 U/ml Penicillin, 0.1 mg/ml streptomycin and 2 mM l-glutamine) containing 10% fetal calf serum, plated at 1 × 10^6^ cells/ml in 2 ml volumes per well in 24-well plates (Appleton woods). Freshly isolated PBMC were cultured for 6 days at 37 °C in the presence of a combination of 1/100,000 standardized pansorbin cells (heat-killed, formalin-fixed *Staphylococcus aureus*, Cowan 1 strain; SAC), 1 μg/ml phosphothiolated CpG oligodeoxynucleotide 2006 (CpG DNA) and 1/1000 pokeweed mitogen extract (PWM). Cells were then harvested and plated at 4 × 10^5^ cells/well on 96-well multiscreen plates (Millipore) pre-coated with a pneumococcal protein antigen (1.5 μg/ml Choline binding protein A (CbpA), 2 μg/ml Pneumococcal surface protein A (PspA), 2 μg/ml Pneumolysin (Ply) or 2 μg/ml Pneumococcal surface antigen A (PsaA); all generated as previously described).^25^ Of the many pneumococcal secreted or surface-expressed proteins, including LytC, PcsB, that have been identified as potential vaccine antigens,^26–29^ work described here was limited to only 4 antigens due to sample constraints. These protein antigens were chosen because they are well characterized as playing a role in the pathogenesis of pneumococcal disease and demonstrated to be protective against carriage and/or invasive disease in humans and/or animal models.^30–33^ Knowledge gained from these 4 protein antigens may be applicable to other novel protein vaccine candidates, as most of these protein antigens are fairly conserved among all pneumococcal isolates. A cocktail of 10 ug/ml tuberculin purified protein derivative (PPD; Statens Serum Ins.), 5 ug/ml purified tetanus toxoid (Merck Biosciences) and 1 ug/ml inactivated split virion influenza vaccine (Flu; Aventis Pasteur MSD) was used as a positive control and PBS alone as a negative control. As PBS generated very few spots, background was not subtracted. As the total number of cells recovered from each blood sample varied, it was not always possible to include all antigens in every assay. ASC were detected by incubation with IgG secondary antibody (Sigma) conjugated to alkaline phosphatase for at least 6 h prior to development with an alkaline phosphatase substrate kit (Bio-Rad). ASC were counted using an AID ELISPOT reader and analysis software version 4.0 (AID Strasburg, Germany).

### Statistics

Data were expressed as medians and interquartile ranges (IQR). Comparisons were made using Friedman's test to evaluate the effect of ART across all time points (i.e. at baseline and all 3 follow-up times). Statistical analysis and graphical presentations were done using Stata 10 and GraphPad Prism software (version 4.0). Differences after comparisons were considered statistically significant if *P* < 0.05.

## Results

### Baseline characteristics of the study participants

Of the 45 children recruited, 2 died, 1 moved away from Blantyre and 1 withdrew from the study. These 4 children were excluded from subsequent analysis and 41 HIV-infected children with median age 92 months at recruitment (IQR, 63–132 months) were included. None of these 41 children was malaria parasitemic at enrollment, all received cotrimoxazole prophylaxis and none were febrile at any of the follow-up visits. 18/41 (44%) were females and 23/40 (58%) had *S. pneumoniae* detected by culture of a nasopharyngeal swab obtained at enrollment. Pneumococcal carriage rates varied between 58 and 92% throughout the course of the study and the rate was 83% after 12 months of ART. The carriage rate in healthy controls with median age 92 months (IQR, 54–132 months) was 46%.^10^

### Changes in lymphocyte subsets

**Figure 1:**
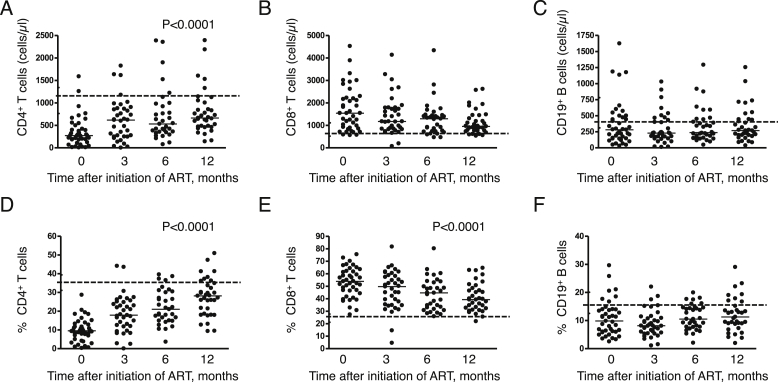
**Progressive changes in major lymphocyte subsets of HIV-infected Malawian children over the course of 12 months' antiretroviral therapy (ART).** Absolute numbers of circulating (**A**) CD4^+^ T cells (**B**) CD8^+^ T cells and (**C**) CD19^+^ B cells; proportions of (**D**) CD4^+^ T cells (**E**) CD8^+^ T cells and (**F**) CD19^+^ B cells measured using flow cytometry. Horizontal bars in A–F represent median values. The broken horizontal lines in A–F represent median values in HIV-uninfected controls recruited as part of separate contemporaneous study reported elsewhere and interquartile range for these controls have been incorporated in the *y*-axis.^10^ Comparisons were made using Friedman's test to evaluate the effect of ART across all time points.

As expected, both absolute and percentage CD4^+^ T cell counts rose significantly (*P* < 0.0001) following initiation of ART over the 12 months of the study. While on average, rises in absolute counts were most obvious during the first 3 months, rises in percentages were more progressive over the whole observation period although in neither case did they reach median values seen in HIV-uninfected controls (Fig. 1A and D). In contrast, no statistically significant trends in absolute CD8^+^ T cell and CD19^+^ B cell counts were seen over the same period (Fig. 1B and C). Values for CD8^+^ T cells remained above those seen in uninfected controls showing some apparent trend towards these normal values (Fig. 1B and E) but median CD19^+^ B cell values remained consistently lower than control values (Fig. 1C and F).

### Changes in B cell subsets

**Figure 2:**
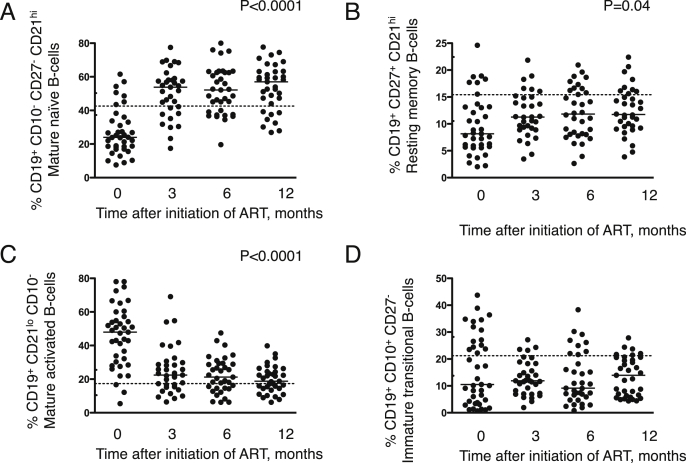
**Increase in mature naïve & resting memory B cells and reduction of apoptosis prone mature activated B cells in HIV-infected children following initiation of antiretroviral therapy (ART)**. Percentages of (**A**) mature naïve B cells (CD19^+^ CD10^−^ CD27^−^ CD21^hi^) (**B**) resting memory B cells (CD19^+^ CD27^+^ CD21^hi^) (**C**) mature activated B cells (CD19^+^ CD21^lo^ CD10^−^) (**D**) immature transitional B cells (CD19^+^ CD10^+^ CD27^−^) measured using flow cytometry in HIV-infected children on ART over 12 months. Horizontal bars in A–D represent median values. The broken horizontal lines in A–D represent median values in HIV-uninfected controls recruited as part of separate contemporaneous study reported elsewhere and interquartile range for these controls have been incorporated in the *y*-axis.^10^ Comparisons were made using Friedman's test to evaluate the effect of ART across all time points.

Extending our recent report of an apoptosis-prone phenotype in HIV-infected children,^10^ we measured trends in circulating B cell subsets during 12 months' ART and observed increases in proportions of both mature naïve (CD19^+^ CD10^−^ CD27^−^ CD21^hi^) and resting memory B cells (CD19^+^ CD27^+^ CD21^hi^) (*P* < 0.0001, *P* = 0.04) which occurred largely over the first 3 months and to levels, in the former subset, that were higher than those seen in uninfected controls while in the latter they remained below normal median values (Fig. 2A–B). There were corresponding falls in proportions of apoptosis-prone mature activated (CD19^+^ CD21^lo^ CD10^−^) B cells (*P* < 0.0001) to levels seen in uninfected controls (Fig. 2C). However, no significant or consistent trends in numbers of apoptosis-prone immature transitional (CD19^+^ CD10^+^ CD27^−^) B cell percentages were observed (Fig. 2D).

### Changes in pneumococcal protein antigen specific immunoglobulin G memory B cells

**Figure 3:**
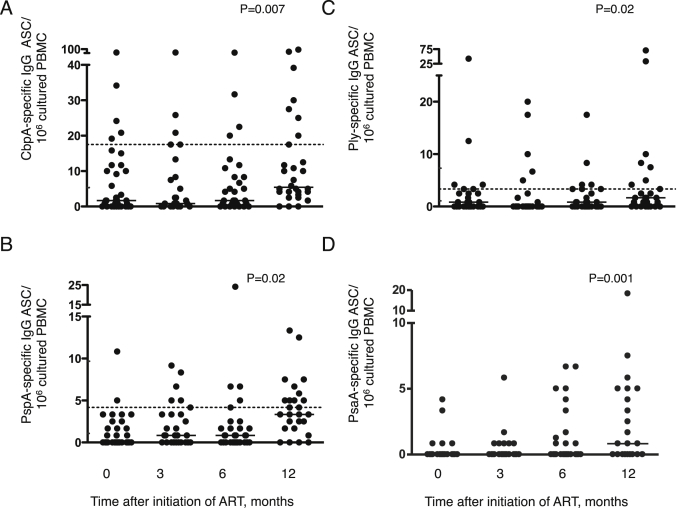
**Pneumococcal protein antigen specific IgG memory B cells increase after 12 months' antiretroviral therapy (ART) in HIV-infected Malawian children.** Using an enzyme-linked immunospot (ELISPOT) assay, following the expansion of memory B cells with a cocktail of mitogens (standardized pansorbin cells, pokeweed mitogen extract and phosphothiolated CpG oligodeoxynucleotide 2006) for six days, (**A**) Choline binding protein A (CbpA), (**B**) Pneumococcal surface protein A (PspA), (**C**) Pneumolysin (Ply), and (**D**) Pneumococcal surface antigen A (PsaA) specific IgG antibody secreting cells (ASCs) were enumerated in HIV-infected children on ART. Memory B cell responses were expressed as numbers of antibody secreting cells (ASCs) per million cultured peripheral blood mononuclear cells (PBMCs) seeded on the ELISPOT well. Each dot represents an average of test wells performed in triplicate. Horizontal bars represent median values. The broken horizontal lines in A–C represent median values in HIV-uninfected controls recruited as part of separate contemporaneous study reported elsewhere.^10^ The interquartile range for these controls has been incorporated in the *y*-axis except the upper quartile band for CbpA specific memory B cells of 44.2 that was not incorporated owing to axis break. PsaA specific memory B cells were not measured in the HIV-uninfected cohort. Comparisons were made using Friedman's test to evaluate the effect of ART across all time points.

In contrast to total B cell subsets, recovery in numbers of circulating memory B cells specific for four pneumococcal antigens (Choline binding protein A (CbpA), Pneumococcal surface protein A (PspA), Pneumolysin (Ply) and Pneumococcal surface antigen A (PsaA)) only became apparent during the latter part of the 12 month observation period (*P* = 0.007, *P* = 0.02, *P* = 0.02, *P* = 0.001 respectively (Fig. 3)). Median values approached those seen in uninfected controls by 12 months for two of the three antigens for which control data were available (Fig. 3).

## Discussion

The reversal of the immunodeficiency, in particular T cell function, associated with untreated HIV and following the initiation of ART is well described.^5,18,23,34–36^ The impact of ART on recovery of B cell function has received less attention. Here we describe reconstitution of B lymphocyte subsets in juxtaposition with reappearance of pneumococcus-specific memory B cells in Malawian children.

Alongside normalization of CD4 and CD8 subsets, correction of B cell subset counts, including mature naïve, resting memory and apoptosis-prone mature activated B cells had largely occurred by 3 months after commencement of ART. In contrast and in the context of prevalent nasopharyngeal pneumococcal carriage throughout the study, circulating pneumococcal protein antigen-specific memory B cells only became more abundant towards the end of the 12-month study period suggesting that antigen-specific B cell memory function may lag behind other indices of B cell recovery.

Although a switch from short-lived apoptosis prone B cells to longer-lived naïve and resting memory B cells was seen relatively early after initiation of ART, there were no significant changes observed in the percentages and absolute numbers of total CD19^+^ B cells during ART in this cohort during the 12 months of observation. Some previous studies in children have shown no changes in total CD19^+^ B cells during ART^37^ while other studies in both adults and children have shown rises.^18,38–40^ There are numerous factors that may differ between these samples which could have contributed to these discordant findings but the most immediate apparent need is to follow these trends over a longer observation period.

We have previously demonstrated acquisition of pneumococcal protein-specific T cell and B cell immunity in both low carriage and high carriage populations.^10,41–45^ This naturally acquired immunity may occur as a result of multiple immunizing carriage events to a range of pneumococcal proteins expressed at the mucosal surface.^46^ In African children, pneumococcal nasopharyngeal carriage occurs very early in life and reaches very high rates^47^ and, as in this study, carriage rates may be higher among those with HIV infection.^48^ In this context, we have recently shown that even in minimally symptomatic HIV-infected children, pneumococcal protein antigen-specific memory B-cell numbers are low compared with HIV-uninfected children.^10^ The delayed recovery in pneumococcal antigen specific B cell memory after institution of ART that we describe here may indicate that these HIV-infected children remain both more vulnerable to invasive pneumococcal disease and to prolonged or higher density carriage. Understanding more clearly how B cell immune reconstitution relates to pneumococcal colonization will be important in countries such as Malawi with high HIV prevalence and where pneumococcal conjugate vaccine (PCV) has recently been introduced into the routine infant immunization schedule, particularly since the effectiveness of these vaccines is to a great extent mediated by effects on carriage and transmission.^49^

Many of the children reported here were commenced on ART with a low percentage CD4 nadir (Fig. 1A). It is possible that the delayed and incomplete restoration of antigen-specific B cell function we observed would have been ameliorated by earlier initiation of ART.^18,50,51^ This question has begun to be tackled programmatically as the thresholds for initiating ART are lowered but merits further investigation if vaccines are to be targeted effectively.

## Funding

This work was supported by the Joan Franklin-Adams Trust (scholarship to O.H.I), David Baum Memorial Appeal (grant to A.F and R.S.H.), MLW Core Programme Grant from the Wellcome Trust (funding to R.S.H. – grant number 084679/Z/08/Z) and a Wellcome Trust project grant (funding to R.S.H. and N.A.W. – grant number 083603/A/07/Z).

## Conflict of interest

A.F. undertakes research, and until October 2014, post-graduate educational and advisory work for Pfizer and GSK who manufacture pneumococcal conjugate vaccines. He receives no personal income for this, all funding being paid to his employers. The other authors have declared that no conflict of interest exists.
